# Peer teaching under pandemic conditions – options and challenges of online tutorials on practical skills

**DOI:** 10.3205/zma001403

**Published:** 2021-01-28

**Authors:** Niklas Julian Dohle, Mareen Machner, Maike Buchmann

**Affiliations:** 1 Universitätsmedizin Berlin, Prodekanat für Studium und Lehre, Lernzentrum, Campus Charité Mitte, Berlin, Germany; 2 Berliner Bildungscampus für Gesundheitsberufe, Bereich Weiterbildung, Berlin, Germany

**Keywords:** clinical competence, medical education, methods, peer group, students, medical, psychology, learning

## Abstract

**Background: **Within days, the corona crisis has forced the “Lernzentrum”, as well as all other places of training and further education, to discontinue classroom teaching at German universities and vocational schools. In order to start teaching online, tutors had to face the challenge to develop new digital learning formats (virtual classrooms) for the peer teaching of practical skills within a short time. This paper aims at outlining the project of developing e-tutorials with regard to the teaching of practical skills.

**Methodology: **After analyzing the classroom lessons (n=30), some of the tutorials were transformed into digital formats. These so-called “e-tutorials” were held via a digital platform. They have been evaluated continuously with a standardized online questionnaire. The results of this evaluation have been analyzed descriptively.

**Results: **From 27/04/2020 to 17/07/2020 eleven different e-tutorial formats were offered on 246 dates. The evaluation revealed a high degree of acceptance with these course offers as well as with the implementation by the tutors.

**Interpretation: **During the pandemic crisis the substitution of peer teaching into forms of e-tutorials was considered valuable; however, these learning formats present challenges, especially with regard to the interaction between teachers and students. They cannot therefore fully replace the peer teaching of practical skills.

## Zusammenfassung

**Hintergrund: **Die Corona-Krise hat das Lernzentrum genauso wie alle anderen Aus- und Weiterbildungsorte an deutschen Universitäten und Berufsfachschulen innerhalb weniger Tage gezwungen, den Präsenzunterricht einzustellen. Die Tutor*innen standen vor der großen Herausforderung, innerhalb von kurzer Zeit neue digitale Lernformate (virtuelle Klassenzimmer) für das Peer-Teaching praktischer Fertigkeiten zu entwickeln und online zu unterrichten. Dieser Beitrag soll das Projekt der Entwicklung von eTutorien bezogen auf praktische Fertigkeiten darlegen.

**Methodik: **Nach einer Prüfung aller Tutorien des Präsenzangebots (N=30) wurde ein Teil der Tutorien in digitale Formate transformiert. Die sogenannten eTutorien wurden live über eine digitale Plattform abgehalten und kontinuierlich mit einem standardisierten Online-Fragebogen evaluiert. Die Evaluationsergebnisse wurden deskriptiv ausgewertet.

**Ergebnisse: **In einem Zeitraum vom 27.04.2020 - 17.07.2020 wurden 11 verschiedene eTutorien-Formate an 246 Terminen angeboten. Die Evaluationen zeigten eine hohe Zufriedenheit mit dem Angebot und der Durchführung durch die Tutor*innen.

**Schlussfolgerung: **Während der Pandemiekrise wurde die Substitution von Peer Teaching in Form von eTutorien als wertvoll erachtet, dennoch bergen diese Lernformate Herausforderungen v.a. bezogen auf die Interaktion und können Peer Teaching von praktischen Fertigkeiten nicht vollständig ersetzen.

## 1. Introduction: peer teaching at the learning center of the Charité

Since the founding of the learning center, the Skills Lab of the Charité, 20 years ago, courses have been offered in peer teaching format. Since 2015, the tutorials have been implemented in the curriculum. All students must participate in a total of 60 teaching units (TU; 1 TU=45 min) of peer teaching in the course of their studies. According to the motto “Learn as you like”, students can choose from a wide range of tutorials offered by various working groups. The majority of the demand is covered by tutorials on practical skills. The team of currently 29 learning center tutors designs the tutorials themselves, the scripts for the implementation are medically approved. Participants particularly praise the learning-friendly atmosphere in the small groups, which is characteristic of peer teaching [1], [2], [3]. 

In view of the corona crisis, the tutorials have been suspended. It became clear soon that a digital offer had to be developed, so-called eTutorials. In the project presented here, the experiences of the tutors and the evaluation of the tutorials by the students are presented in comparison to the previous semester. The first 14 weeks of the semester are compared, in the winter semester 2019/20 (tutorials exclusively in attendance) the period from 14/10/2019-1/01/2020, in the summer semester 2020 (eTutorials) from 27/04/2020-17/07/2020.

## 2. Project description: development of eTutorials on practical skills

There are several ways to move lessons from the “normal” to the virtual classroom. Within the Charité, Microsoft Teams [https://www.office.com/] is used, a software that allows video conferences with parts of the screen and the integration of various other applications. 

In a first step, the presence offer was examined with the question of which tutorials can be digitally transformed into practical skills. Criteria for this were, among other things, whether the practical skills could be presented well in videos and whether the production of these videos was possible in the pandemic situation. For example, no videos with mutual examinations were recorded. Tutorials with a seminaristic character, such as “Chest X-ray”, were implemented online. After an adaptation of first tutorials, the implementation of eTutorials was started in early May 2020. As usual, the tutorials were booked via the Charité’s own online platform.

Each tutorial was followed by a voluntary standardized online evaluation with EvaSys^©^ [https://www.evasys.de/]. All participants were presented with statements on the quality of the implementation, process and results and were asked about their degree of agreement (1=fully agree/5=disagree) using a five-level Likert scale. The questionnaires also contained free text fields in which praise and suggestions for improvement could be expressed.

## 3. Results: offer and evaluation

**Figure 1:**
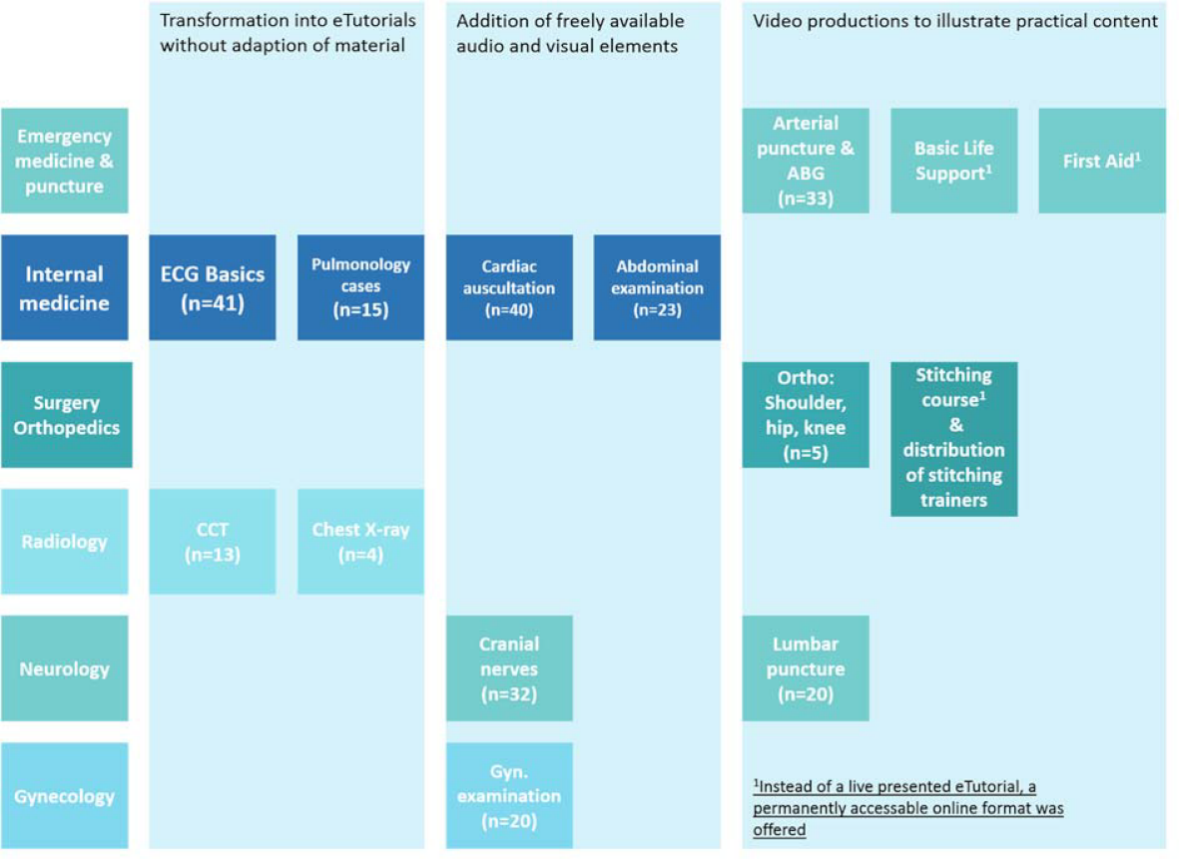
Illustration of different ways to implement eTutorials

**Figure 2:**
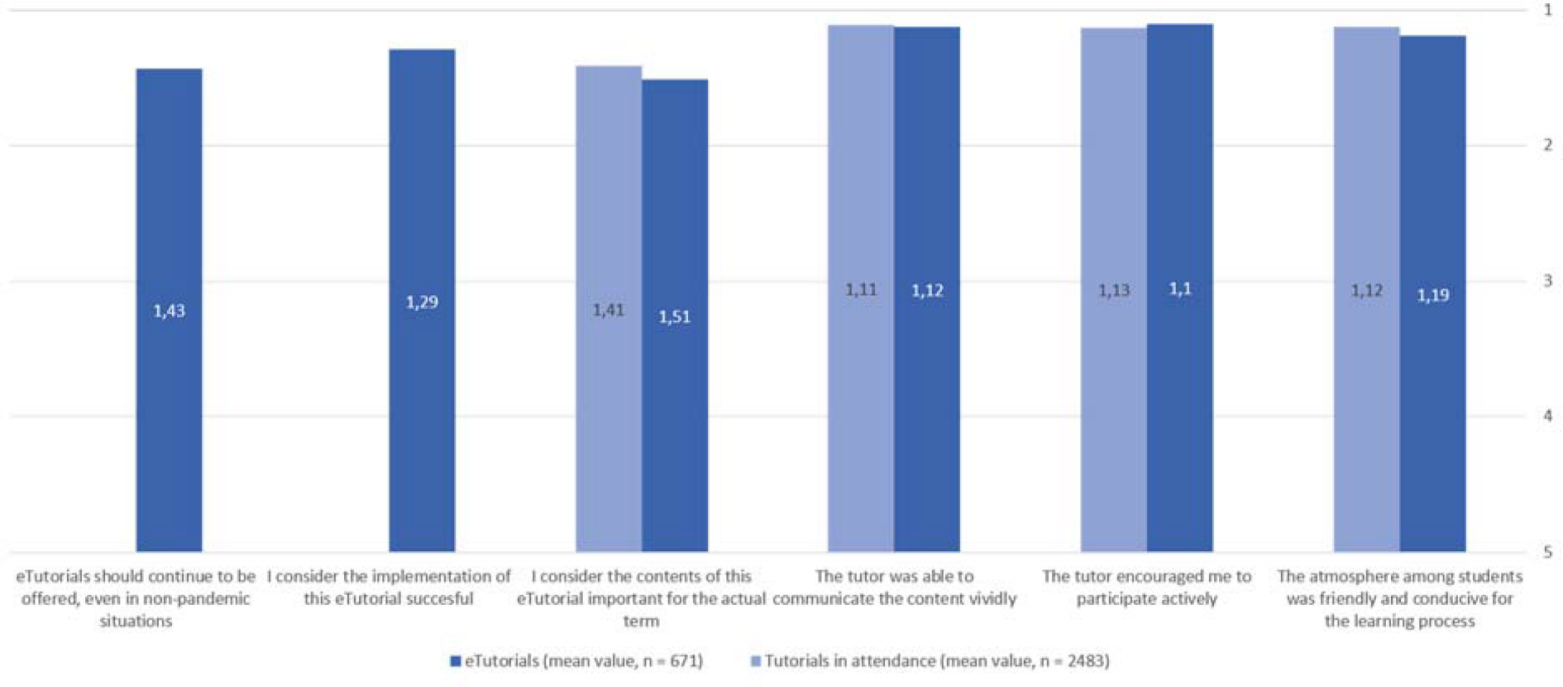
Comparison of the evaluation results

In a period from 27/04/2020-17/07/2020 eleven different eTutorial formats (see table 1 (Tab. 1) and figure 1 (Fig. 1)) were offered on 246 dates. The evaluation showed a high level of satisfaction with the offer and the implementation by the tutors (see figure 2 (Fig. 2)). 

In the free text fields, the videos as well as interactive learning formats, such as a quiz on an app, and the practice on clinical case studies were positively highlighted as practical application. 

Disadvantages due to technical problems on the student side were rather rare and were only explicitly mentioned in the evaluations in connection with the embedding of audio tracks in a presentation split across the screen (see table 2 (Tab. 2)).

## 4. Discussion

This paper describes the development of eTutorials into practical skills at the learning center. While in the evaluations the participants' agreement with an encouragement to participate was similarly high as in the face-to-face tutorials, from the tutors’ point of view there were significantly greater difficulties in activating the participants in eTutorials. 

From the tutors’ point of view, a small group size (max. 4-8 persons) and the fact that all participants switched on the video function were considered helpful. The use of method changes and especially the quiz function has proven to be a playful approach to activation [4], [5]. The tutors were able to expand their didactic skills in the Corona pandemic by designing virtual lessons, skills that will become increasingly important for teachers in the future, even regardless of current restrictions [6].

Overall, participants and tutors alike are in favor of offering eTutorials beyond the pandemic restrictions, as they ensure a high degree of flexibility. 

Although videos, for example, can support the learning of practical skills [7], [8], it is undisputed that practical skills cannot be learned completely in an online format. The eTutorials could be offered as a preliminary course in the sense of blended learning in order to use the time in presence even more efficiently for guided practice. Despite the short-term changeover, almost 60% of the teaching units could be credited as eTutorials compared to the same period in the previous semester. However, the range of courses offered included significantly fewer different formats, so that the choice of tutorials for students was limited in the sense of self-determined learning. Technical problems played a minor role [9]. Although the data collected provided a basis for discussion, the surveys are to be conducted on further and larger populations and a longer intervention period. A further bias probably results from the composition of tutors and students who are exclusively assigned to a university. Therefore, it is to be expected that the results are only partially transferable to other universities.

## 5. Conclusion

- Peer Teaching can also create an atmosphere conducive to learning at a distance.
- eTutorials cannot and will not replace peer teaching of practical skills.
- At the same time, eTutorials in and outside of pandemic times will continue to be a useful addition in the future.
- eTutorials present teachers and learners with new pedagogical challenges.

## Competing interests

The authors declare that they have no competing interests. 

## Figures and Tables

**Table 1:**
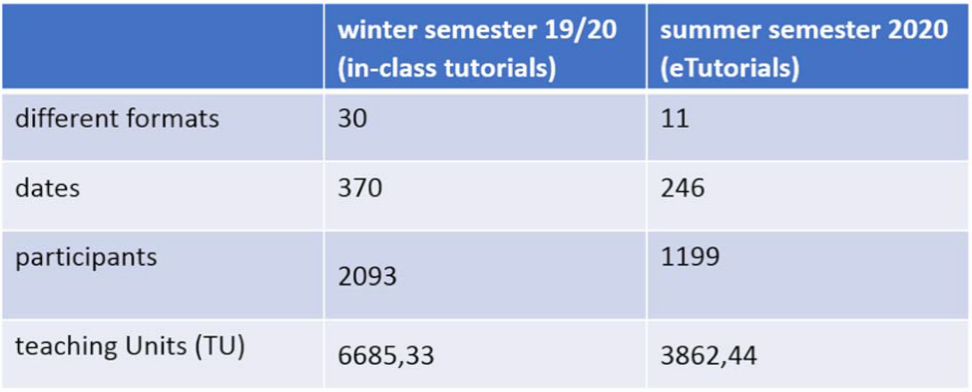
Presentation of the tutorial offer in temporal comparison

**Table 2:**
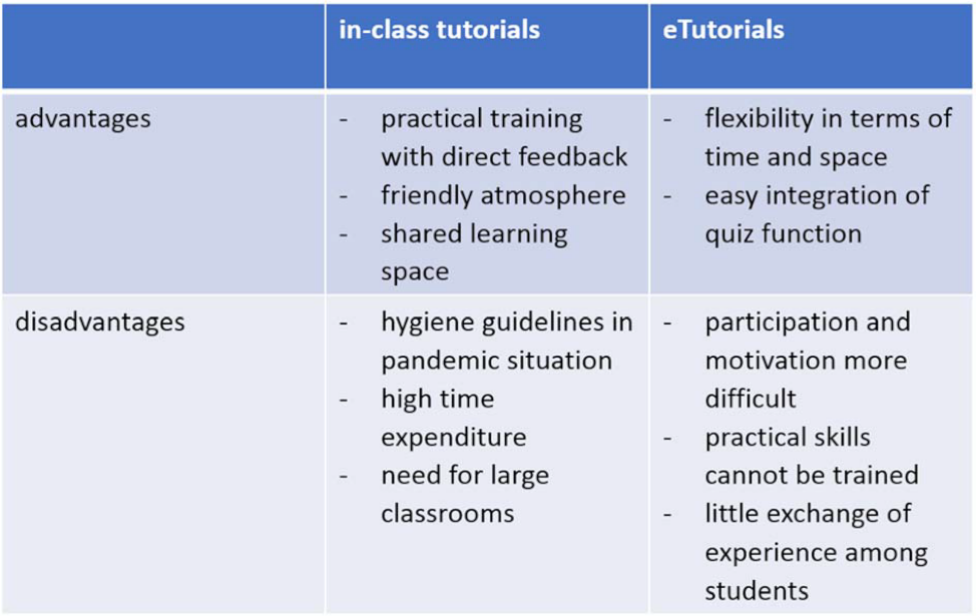
Advantages and disadvantages of in-class turorials and eTutorials
